# Short and Long-Term Mortality Trends for Cancer Patients with Septic Shock Stratified by Cancer Type from 2009 to 2017: A Population-Based Cohort Study

**DOI:** 10.3390/cancers13040657

**Published:** 2021-02-06

**Authors:** Youn-Jung Kim, Min-Ju Kim, Ye-Jee Kim, Won Young Kim

**Affiliations:** 1 Department of Emergency Medicine, University of Ulsan College of Medicine, Asan Medical Center, Seoul 05505, Korea; yjkim.em@gmail.com; 2 Department of Clinical Epidemiology and Biostatistics, Asan Medical Center, Seoul 05505, Korea; minjukim@amc.seoul.kr (M.-J.K.); kimyejee@amc.seoul.kr (Y.-J.K.)

**Keywords:** septic shock, neoplasms, mortality, trends, epidemiology

## Abstract

**Simple Summary:**

This study aimed to assess short and long-term mortality trends in cancer patients with septic shock from 2009 to 2017. Among 43,466 adult cancer patients with septic shock (90% solid and 10% hematologic cancer cases) who presented at an emergency department (ED) in Korea between 2009 and 2017, the 30-day and 1-year mortality rates were 52.1% and 81.3%, respectively. The overall 30-day mortality decreased by 4.8% annually from 2013 to 2017, whereas the 1-year mortality only showed a 1.9% annual decrease over this same period. Pancreatic cancer cases showed the most significant improvement in the 30-day mortality since 2014, and lung and stomach cancer showed a sustained decrease in this metric during the whole study period. The outcomes of cancer patients with septic shock have improved in recent years across most cancer types. Physicians should have expectations of improved prognoses in cancer patients admitted to the ED with septic shock.

**Abstract:**

There have been recent advances in both cancer and sepsis management. This study aimed to assess short and long-term mortality trends in cancer patients with septic shock from 2009 to 2017 by cancer type. This nationwide population-based cohort study using data from the National Health Insurance Service of Korea included adult cancer patients who presented to an emergency department (ED) with septic shock from 2009 to 2017. Among 43,466 adult cancer patients with septic shock (90% solid and 10% hematologic cancer cases), the 30-day and 1-year mortality rates were 52.1% and 81.3%, respectively. The overall 30-day mortality showed a marked decrease of 4.8% annually from 2013 to 2017, but the annual decrease in the 1-year mortality over the same period was only 1.9%. Pancreatic cancer cases showed the most significant improvement in 30-day mortality between 2014 and 2019 (11.0% decrease/year). Lung and stomach cancers showed a sustained decrease in 30-day mortality during the whole study period (1.7% and 2.0% decrease/year, respectively). The outcomes of cancer patients with septic shock have improved in recent years across most cancer types. Physicians should have expectations of an improved prognosis in cancer patients admitted to the ED with septic shock.

## 1. Introduction

Cancer is a global health burden with an increased incidence that is estimated to now be 20% in people aged 75 years and older [1]. The major advances that have emerged in the diagnosis and treatment of cancer have improved the survival outcomes over the past two decades [2,3,4,5,6], but critical complications increase with a longer duration of these diseases, consistent with the organ dysfunction that can arise from both the progression of cancer and from treatments such as chemotherapy, radiotherapy, and surgery [7,8,9]. Septic shock is one of the most common life-threatening complications in cancer patients, which shows a high mortality rate [6,10,11,12,13].

Although a preexisting diagnosis of cancer was known as an independent risk factor of death from sepsis in patients admitted to the intensive care unit (ICU) [14,15], the survival outcome in cancer patients with sepsis has been improved over time [6,16,17]. However, cancer patients who develop septic shock still pose a difficult dilemma for clinicians in terms of use of resources and costs of the health care, especially in the emergency department (ED) setting [16]. Minimal reliable data are available to guide the physicians, cancer patients, and their families in their medical decision-making, and better information to assist with this is therefore urgently needed [16,17]. Moreover, recent improvements in oncology care and early protocol-driven resuscitation bundled with therapy for sepsis are likely to have contributed to the more favorable outcomes in cancer patients with septic shock, so there is a need to reevaluate the prognosis in these cases.

ED utilization by cancer patients has increased [18], however, there remains a paucity of data on the proportion of the cancer patients who present to the ED with septic shock, particularly in relation to the types of malignancy. Therefore, an enhanced understanding of the outcome trends in the cancer patients who present to the ED with septic shock will help to establish better treatment strategies in terms of the selection of suitable patients as well as allocation of clinical resources. The objective of our present population-based study was to assess the short and long-term mortality trends in Korean cancer patients with septic shock who visited an an ED between 2009 and 2017, stratified across cancer types.

## 2. Materials and Methods

### 2.1. Study Design and Data Source

This was a population-based cohort study using the data from the Korean National Health Information Database (NHID) that was collected between 2009 and 2017 and was released in 2019. The National Health Insurance Service (NHIS) was launched as the single insurer covering all Korean citizens through the enactment of the Medical Insurance Act in 1963 [19]. The Korean NHIS is responsible for maintaining and managing the NHID, which is a public database covering health care utilization, health screening, socio-demographic variables, and mortality for all Korean citizens [19]. The data we extracted included demographic information, medical bill details, medical treatments, disease histories, and prescriptions, which were converted as insurance claim information for the first day of medical treatment. The prescription information in NHID included prescription drugs (according to the NHIS formulary code), prescription dates, dose, supply days, and administration routes. Laboratory and radiologic data were not available from NHID.

The primary outcome of this present study was all-cause 30-day mortality, and the secondary outcome was all-cause 1-year mortality. The cancer patients with septic shock in our study population were followed up with from the index date to 1 year, or until the date of death if it occurred before 1 year. The study was approved by the Institutional Review Board of the Asan Medical Center (Study number: 2019-0743) and by the NHIS inquiry commission. The personal privacy of the study subjects was protected through the de-identification of the national insurance claims data.

### 2.2. Study Patients and Data Definitions

We selected all patients admitted to a hospital via the ED from 2009 to 2017 and who fulfilled the clinical surveillance definition of septic shock. The Third International Consensus Definitions for Sepsis and Septic Shock (Sepsis-3) define septic shock as “life-threatening organ dysfunction caused by a dysregulated host response to infection, requiring vasopressor therapy, and known elevated lactate level.” [20]. We used a clinical surveillance definition of septic shock, based on concurrent vasopressors, antibiotics, and blood cultures [21]. Among the patients with a blood culture order and the concomitant administration of intravenous antibiotics (suspected infection), those who received any type of vasopressor including dopamine, norepinephrine, epinephrine, vasopressin, and phenylephrine were defined as the septic shock cases.

Patients with cancer were identified within the initial screened cohort as those having an in-patient or out-patient visit with a cancer diagnosis code within the preceding 90 days of their septic shock hospitalization, in accordance with the International Classification of Diseases, 10th edition (ICD-10) and rare incurable disease registration code (V193, V027) simultaneously to minimize misclassification. Since 2005, the Korean NHIS has covered 95% of the total medical expenses for any healthcare services related to cancer treatment for 5 years after diagnosis. The Health Insurance Review and Assessment Service (HIRA) of South Korea evaluates the adequacy of healthcare services and medical fee claims. When HIRA confirms an inadequate medical charge related to healthcare services, NHIS cancels the payment or demands health care providers to pay back the medical payment by NHIS. The cancer registration program for financial support and scrutiny of the NHIS enhanced the registration of the cancer diagnosis codes as either a principal or secondary diagnosis in Korean cancer patients [22]. In addition, the accuracy of identifying cancer patients using the combination of ICD-10 codes (C00–C97) and cancer registration code (V193, V027) in NHID was similar to that of the Korea National Cancer Incidence Database, estimated to be 98.2% complete [23,24]. The primary cancer sites in our current study cohort were categorized into 21 types according to the categorization from the Korean Cancer Association [24]. Underlying comorbidities were identified using the ICD-10 codes when two or more hospital visits with the relevant diagnostic codes within a year prior to the septic shock date were recorded and the Charlson Comorbidity Index (CCI) was calculated [25]. Patients who were less than 18 years old at the time of their septic shock hospitalization or patients without full data were excluded. In cases of patients admitted more than once because of septic shock, we used data collected at the first hospitalization.

### 2.3. Statistical Analysis

Descriptive analyses were conducted to evaluate the characteristics of the 30-day survivors and non-survivors among the current study patients. Hazard ratios (HRs) and 95% confidence intervals (CIs) for all-cause 30-day mortality were estimated using Cox proportional hazard regression analyses. After adjustment for age, sex, and CCI, adjusted HRs of the hospitalization year on 30-day mortality were calculated. We used the Joinpoint regression model to examine the mortality rate trends over the 9-year study period and also calculated these trends by predominant cancer type. A Joinpoint regression was estimated for the eight predominant cancer types by using the Joinpoint software, Version 4.5.0.1 (Statistical Research and Applications Branch, National Cancer Institute) to identify changes in the mortality rate trends [26]. The join point regression identifies the time point of a trend change and calculates the annual percentage change in rates between trend-change years, respectively. The average annual percentage change across the whole study period was also calculated. All tests of significance used two-sided *p* values at less than 0.05. These analyses were conducted using Enterprise Guide version 7.1 (SAS Institute Inc., Cary, NC, USA).

## 3. Results

**Figure 1:**
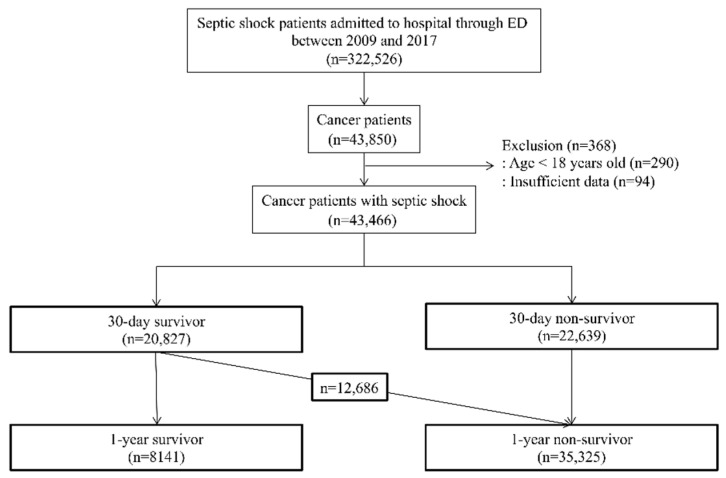
Flow diagram for the study cohort derivation from the Korean National Health Information Database. ED indicates emergency department.

From 2009 to 2017, 322,526 hospitalized patients in Korea through the ED met the clinical criteria for septic shock, and 43,850 (13.6%) of these patients had ICD-10 codes for cancer (C00–C97) and a cancer registration code (V193, V027) within the preceding 90 days of their septic shock hospitalization (Figure 1). Patients who were less than 18 years old at the time of their septic shock hospitalization (*n* = 290) or patients without full data (*n* = 94) were excluded. Finally, 43,466 patients were included in our current analyses, and 22,639 (52.1%) of these cases died within 30 days of their hospitalization.

### 3.1. Characteristics of the Cancer Patients with Septic Shock Included in this Study

Table 1 presents the characteristics of the current study patients according to their 30-day mortality outcomes. Patients aged 60 years and above accounted for 74.0% of the population, and age showed a graded association with the 30-day mortality by univariate Cox-Proportional analysis. The CCI showed a graded association with the 30-day mortality. Liver cirrhosis (11.4%) showed the highest HR of 1.364 (95% CI, 1.313–1.417; *p* < 0.001), followed by chronic lung disease (HR, 1.212; 95% CI, 1.166–1.260; *p* < 0.001).

The 30-day mortality rate was further found to significantly vary by cancer type in our current cohort. Among the 21 cancer types in the study population, 4 predominant types accounted for nearly half (*n* = 21,073, 48.5%) of the study patients, i.e., lung (*n* = 6657, 15.3%), liver (*n* = 6238, 14.4%), colon (*n* = 4494, 10.3%), and stomach (*n* = 3684, 8.5%). The two highest HRs for 30-day mortality were lung (HR, 2.292; 95% CI, 2.028–2.591; *p* < 0.001) and liver (HR, 2.160; 95% CI, 2.909–2.442; *p* < 0.001) cancers.

### 3.2. General Trends in the 30-Day and 1-Year Mortality Outcomes among the Cancer Patients with Septic Shock

**Figure 2:**
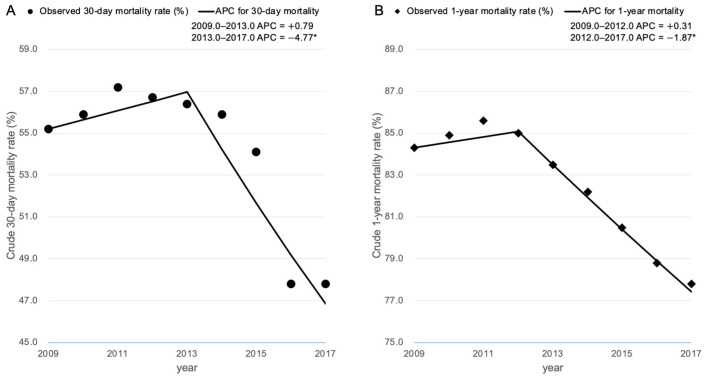
Join point regression analysis of mortality trends among adult active cancer patients with septic shock in Korea between 2009 and 2017. (**A**) 30-day mortality. (**B**) 1-year mortality. * Significantly different from zero (*p* < 0.05). APC indicates annual percentage change.

The proportions of the seven predominant cancer types (5 for solid cancers and 2 for hematologic cancers) in our present cohort showed no substantial change over time (Figure S1). During the 9-year study period, the number of cancer patients with septic shock increased from 3987 in 2009 to 11,592 in 2017. The 30-day mortality rate decreased from 55.2% in 2009 to 47.8% in 2017 (absolute decrease, 7.4%; *p* for trends < 0.001) (Figure 2A). Our join point regression analysis for 30-day mortality found a join point in 2013, leading to two periods with a different trend, i.e., the late period (2013/2017) showing a 4.77% annual decrease in mortality and an early period (2009/2013) showing no significant change but with an increase of 0.79% in the 30-day mortality. The 1-year mortality also declined from 84.3% in 2009 to 77.8% in 2017 (absolute decrease, 6.5%; *p* for trends < 0.001) and one joint point was identified in 2012; there was an almost level trend in 2009/2012 and a slight decrease in 2012/2017 (0.31% and −1.87% annual change, respectively) (Figure 2B).

After adjusting for age, sex, and CCI, the hospitalization year was found to be significantly associated with the 30-day mortality in our cancer patients with septic shock (Table 2). Compared to the 2009 hospitalization year, there was significant improvement in the 30-day mortality in the patients hospitalized in 2016 (adjusted HR, 0.793; 95% CI, 0.754–0.835; *p* < 0.001) and 2017 (adjusted HR, 0.793; 95% CI, 0.754–0.835; *p* < 0.001).

### 3.3. Trends in the 30-Day and 1-Year Mortality among the Cancer Patients with Septic Shock Stratified by Cancer Type

Table 3 summarizes the 30-day and 1-year mortality trends among our current study patients with septic shock, stratified by specific cancer type. The average annual percentage change between 2009 and 2017 indicated that both the 30-day and 1-year mortality had decreased for most cancer types, but that these declining trends were not evident for the 1-year mortality outcomes. The lung and stomach cancer cases showed no join point in either the 30-day or 1-year mortality, with significant improvement seen in mortality during the overall study period (average decline of 1.7% and 2.0% decrease/year, respectively). A join point was identified for 30-day mortality in the hepatobiliary, colon, and pancreatic cancer cases. The colon and pancreatic cancer patients demonstrated significant improvements in their 30-day mortality outcomes in the period after a join point. Pancreatic cancer showed the highest declining mortality rate between 2014 and 2019 (an average decline of 11.0% decrease/year), followed by colon cancer between 2011 and 2019 (an average decline of 5.0% decrease/year). In contrast to solid cancers, leukemia and non-Hodgkin’s lymphoma cases showed a trend toward an improved 30-day mortality but without statistical significance.

## 4. Discussion

Our current national population-based cohort study found that both the 30-day and 1-year mortality rates among cancer patients with septic shock had an overall improvement between 2009 and 2017. The 30-day mortality rate in this population significantly decreased by 4.8% annually from 2013 (from 56.4% in 2013 to 47.8% in 2017; absolute decrease, 8.6%). Lung and stomach cancers showed a continuous 30-day mortality improvement with 1.7% and 2.0% annual reductions, respectively, over the 9-year study period, whilst pancreatic cancer cases showed the biggest improvement in the 30-day mortality rate with an 11% annual reduction between 2014 and 2019.

A previous study reported an upward trend in the incidence and downward trend in the mortality rate for sepsis and septic shock [27]. A recent study using the sepsis diagnosis code reported that the incidence of sepsis increased from 173.8 to 233.6 per 100,000 population between 2007 and 2016, and the in-hospital mortality decreased from 30.9% to 22.6% in the same period [28]. Notably, however, previous study findings would be altered if the new consensus septic shock definition suggested by Sepsis-3 was applied [20]. In addition, ascertainment bias is prone to occur when using diagnosis code data to identify sepsis and septic shock trends [21,29]. Kadri et al. demonstrated the different rising incidences of septic shock at 27 academic hospitals in the United States from 2005 to 2014 using clinical surveillance data (from 12.8 to 18.6 per 1000 hospitalizations, average 4.9% increase/year) versus claim data (from 6.7 to 19.3 per 1000 hospitalizations, average 19.8% increase/year), and suggested that clinical surveillance definitions for septic shock were superior for identifying the septic shock patients through a clinical medical record review [27]. To the best of our knowledge, our present report is the first nationwide study of the 30-day and 1-year mortality rate trends over a long period in cancer patients with septic shock that has used these more reliable clinical surveillance definitions [27]. Consistent with previous studies, we found from our present analyses that the number of cancer patients who presented with septic shock more than doubled from 3987 in 2009 to 11,592 in 2017. This increase was likely due to not only better recognition with more aggressive treatment and an actual increase in sepsis, the commonly suggested reasons in previous epidemiologic studies, but also because of an increase in cancer prevalence [7,24,28,30].

More than half of the cancer patients in our current series (52.1%) who presented with septic shock at the ED died within 30 days. Interestingly, however, the 30-day mortality trend in our current cohort showed a significant 4.8% annual decrease after 2013, i.e., from 56.4% in 2013 to 47.8% in 2017. No clear difference was identified between 2009 and 2013 (55.2% in 2009 and 56.4% in 2013). In addition, the huge improvement in the 30-day mortality rate should be noted from 54.1% in 2015 to 47.8% in 2016. The changes in the medical environment that took place between 2013 and 2016 offer some clues to explain why cancer patients with septic shock had improved outcomes. Of note in this regard, the Ministry of Health and Welfare of the Korean Government announced that the government would focus to improve the treatment of critically ill patients in 2013, and a hospital quality assessment that includes the intensivist-to-patient ratio and the nurse-to-patient ratio for the ICU has been implemented since 2014 biennially. Moreover, the Ministry of Health and Welfare of the Korean Government amended Article 26–1 of the Act on emergency medical services in 2015, which requested that level I and II emergency centers expand their facilities and medical personnel to increase the treatment capacity and quality for critically ill patients, and an emergency center quality assessment has also been implemented. NHIS pays differently for medical costs per the grades of the hospital’s and emergency center’s quality assessments. In addition, the Korean Shock Society was established in 2013 and consists of emergency physicians and intensivists to research and improve the treatment of shock [31]. Korean hospitals and emergency centers may have started to increase the number of intensivists or providers based on the policy changes. Our findings of improving short-term and long-term mortalities between 2013 and 2017 and a great reduction of 30-day mortality between 2015 and 2016 in cancer patients with septic shock are consistent with a previous study about the incidence and clinical outcomes of hospitalized patients with a diagnosis code of sepsis in Korea using data from NHID that demonstrated a downward trend in the in-hospital mortality rate and a big improvement in the in-hospital mortality rate from 26.1% in 2015 to 22.6% in 2016 [28].

In the early 2000s, an ICU admission of cancer patients with septic shock was thought to be a medically futile effort. However, the consensus on this has changed over time in relation to such ICU care for cancer patients, and it is now considered that the outcomes for cases with and those without cancer could be similar with appropriate patient selection [16,17,32]. Such changes in the ICU treatment approaches for cancer patients with septic shock have also encouraged more aggressive interventions and led to improved outcomes. Our current study found that 1-year mortality rates also had a sustained decrease over time, which indicates that survival after recovery from septic shock has improved for these cases. Such improvement of 1-year mortality could be interpreted as a contribution of the following: (1) advances in cancer treatment regimens; (2) the increased detection of patients with early-stage cancer from a national cancer screening program, and (3) enhanced sepsis management [24,33,34]. The evolution in cancer treatment in addition to enhanced sepsis management has also contributed to the improvement in both short-term and long-term outcomes of cancer patients with septic shock [30].

We also investigated the 30-day and 1-year mortality rates in our present study by specific cancer type, and the trends in this regard also suggested that advances in sepsis care and cancer treatments had improved patient outcomes. The 30-day mortality rates in the pancreatic cancer patients in our series with septic shock demonstrated a remarkable improvement with an 11% annual reduction between 2014 and 2019, although the 1-year mortality showed no significant changes over time in these cases. These findings are consistent with the cancer statistics in Korea showing that survival rate improvements from 1993 to 2017 have been the slowest for pancreatic cancer [24]. In contrast to our pancreatic cancer cases, both the lung and stomach cancer patients in our series showed continuous improvement in both 30-day and 1-year mortality rates over the study period, which is again consistent with the cancer statistics in Korea that have shown outstanding improvements in survival rates for these cancer types [24]. The mortality rates associated with hematologic cancers showed a tendency to decrease but this was without significance. These different trend patterns for various cancer types suggest that improved outcomes can continue to occur in cancer patients with septic shock as a result of advances in cancer treatment. On the other hand, advances in sepsis care would be expected to have a positive impact on short-term outcomes and all cancer patients with septic shock are likely to benefit from early and aggressive ICU care regardless of the cancer type [6,35,36,37].

Our study had some notable strengths and weaknesses. Our utilization of a nationwide database with high coverage of the population (97%), and the fact that our study population contained very recent cases identified by objective clinical surveillance criteria for septic shock using the updated definition of sepsis-3, are strengths of our study design that will help to provide a more comprehensive understanding of cancer patients with septic shock. However, the National Health Information Database in Korea did not provide specific laboratory and clinical data, both of which could have affected the outcomes. It was also not possible to distinguish the cancer stage, treatment setting, or performance status from the diagnosis codes. Moreover, the lack of the specific management data such as the timing of fluid, antibiotic, and vasopressor administration was another limitation of this study. Finally, the patient’s ethnicity is known to be significantly associated with cancer prevalence, which limits the general applicability of our study as the subjects were almost all Asian.

## 5. Conclusions

The septic shock-associated mortality rate in cancer patients has started to decline in recent years across almost all cancer types. Physicians should be able to expect improved prognoses in cancer patients with septic shock and should place an increased emphasis on sepsis management rather than on the cancer in these cases.

## Figures and Tables

**Table 1:** Characteristics and hazard ratios for 30-day mortality among the study subjects.

Characteristic	Total (*n* = 43,466)	Survivor (*n* = 20,827)	Non-Survivor (*n* = 22,639)	Univariate Analysis		
				Hazard Ratio	95% Confidence Interval	*p* Value
Age, years						
18–29	397 (0.9%)	256 (1.2%)	141 (0.6%)	Reference		<0.001
30–39	828 (1.9%)	463 (2.2%)	365 (1.6%)	1.344	1.106–1.632	0.003
40–49	2786 (6.4%)	1408 (6.8%)	1378 (6.1%)	1.576	1.325–1.874	<0.001
50–59	7300 (16.8%)	3633 (17.4%)	3667 (16.2%)	1.630	1.378–1.929	<0.001
60–69	11,772 (27.1%)	5875 (28.2%)	5897 (26.1%)	1.634	1.382–1.931	<0.001
70–79	13,576 (31.2%)	6187 (29.7%)	7389 (32.6%)	1.841	1.559–2.175	<0.001
≥80	6807 (15.7%)	3005 (14.4%)	3802 (16.8%)	1.909	1.614–2.259	<0.001
Female	15,399 (35.4%)	7736 (37.1%)	7663 (33.9%)	0.909	0.884–0.934	<0.001
Comorbidities						
Hypertension	23,136 (53.2%)	10,790 (51.8%)	12,346 (54.5%)	1.078	1.050–1.107	<0.001
Diabetes	16,977 (39.1%)	7828 (37.6%)	9149 (40.4%)	1.079	1.051–1.108	<0.001
Congestive heart failure	5745 (13.2%)	2540 (12.2%)	3205 (14.2%)	1.123	1.082–1.166	<0.001
Chronic lung disease	5008 (11.5%)	2084 (10.0%)	2924 (12.9%)	1.212	1.166–1.260	<0.001
Renal failure	2783 (6.4%)	1262 (6.1%)	1521 (6.7%)	1.071	1.017–1.129	0.009
Liver cirrhosis	4974 (11.4%)	1900 (9.1%)	3074 (13.6%)	1.364	1.313–1.417	<0.001
Charlson comorbidity index						
0–2	7816 (18.0%)	4731 (22.7%)	3085 (13.6%)	Reference		<0.001
3–4	9652 (22.2%)	4922 (23.6%)	4730 (20.9%)	1.366	1.306–1.430	<0.001
5–7	8256 (19.0%)	3986 (19.1%)	4270 (18.9%)	1.469	1.402–1.538	<0.001
≥8	17,742 (40.8%)	7188 (34.5%)	10,554 (46.6%)	1.822	1.750–1.896	<0.001
Cancer type						
Brain	772 (1.8%)	500 (2.4%)	272 (1.2%)	Reference		<0.001
Lung	6657 (15.3%)	2469 (11.9%)	4188 (18.5%)	2.292	2.028–2.591	<0.001
Liver	6238 (14.4%)	2506 (12.0%)	3732 (16.5%)	2.160	1.909–2.442	<0.001
Colon	4494 (10.3%)	2611 (12.5%)	1883 (8.3%)	1.310	1.154–1.488	<0.001
Stomach	3684 (8.5%)	1819 (8.7%)	1865 (8.2%)	1.677	1.477–1.905	<0.001
Gall bladder	1981 (4.6%)	1117 (5.4%)	864 (3.8%)	1.336	1.166–1.532	<0.001
Pancreas	1943 (4.5%)	913 (4.4%)	1030 (4.6%)	1.782	1.559–2.037	<0.001
Leukemia	1917 (4.4%)	864 (4.2%)	1053 (4.7%)	1.822	1.594–2.082	<0.001
Non-Hodgkin’s lymphoma	1475 (3.4%)	742 (3.6%)	733 (3.2%)	1.583	1.378–1.820	<0.001
Female reproductive system	1249 (2.9%)	740 (3.6%)	509 (2.3%)	1.281	1.106–1.485	0.001
Breast	1112 (2.6%)	574 (2.8%)	538 (2.4%)	1.623	1.402–1.877	<0.001
Kidney/bladder	1095 (2.5%)	567 (2.7%)	528 (2.3%)	1.556	1.344–1.801	<0.001
Multiple myeloma	923 (2.1%)	446 (2.1%)	477 (2.1%)	1.712	1.475–1.987	<0.001
Male reproductive system	754 (1.7%)	356 (1.7%)	398 (1.8%)	1.817	1.558–2.120	<0.001
Oropharynx	439 (1.0%)	242 (1.2%)	197 (0.9%)	1.458	1.214–1.751	<0.001
Esophagus	391 (0.9%)	190 (0.9%)	201 (0.9%)	1.681	1.401–2.017	<0.001
Thyroid	169 (0.4%)	101 (0.5%)	68 (0.3%)	1.220	0.936–1.592	0.14
Larynx	149 (0.3%)	82 (0.4%)	67 (0.3%)	1.399	1.071–1.827	0.01
Hodgkin lymphoma	50 (0.1%)	23 (0.1%)	27 (0.1%)	1.642	1.106–2.439	0.01
Other, unspecified	2995 (6.9%)	1622 (7.8%)	1373 (6.1%)	1.442	1.266–1.642	<0.001
Multiple	4979 (11.5%)	2343 (11.3%)	2636 (11.6%)	1.788	1.578–2.026	<0.001

**Table 2:** Impact of hospitalization year among the cancer patients with septic shock on the 30-day mortality, adjusted for age, sex, and Charlson comorbidity index using multivariate Cox-proportional hazard analysis.

Characteristics	Adjusted Hazard Ratio	95% Confidence Interval	*p* Value
Hospitalization year			
2009	Reference		<0.001
2010	0.995	0.938–1.054	0.86
2011	1.029	0.970–1.092	0.34
2012	1.011	0.952–1.074	0.72
2013	1.026	0.962–1.095	0.44
2014	1.026	0.958–1.099	0.46
2015	0.964	0.898–1.035	0.31
2016	0.793	0.754–0.835	<0.001
2017	0.788	0.750–0.828	<0.001
Age, years			
18–29	Reference		<0.001
30–39	1.245	1.025–1.512	0.03
40–49	1.406	1.182–1.672	<0.001
50–59	1.437	1.214–1.701	<0.001
60–69	1.418	1.200–1.677	<0.001
70–79	1.640	1.388–1.938	<0.001
≥80	1.845	1.560–2.184	<0.001
Female	0.910	0.885–0.935	<0.001
Charlson comorbidity Index			
0–2	Reference		<0.001
3–4	1.373	1.312–1.437	<0.001
5–7	1.455	1.389–1.524	<0.001
≥8	1.861	1.787–1.938	<0.001

**Table 3:** Trends in the 30-day and 1-year mortality outcomes by cancer type among the cancer patients with septic shock.

Cancer Type	Period	30-Day Mortality			Period	1-Year Mortality		
		Change Year	APC (95% CI)	AAPC (95% CI)		Change Year	APC (95% CI)	AAPC (95% CI)
Lung cancer	2009–2017	None	−1.7 (−2.7, −0.7) ^1^	−1.7 (−2.7, −0.7) ^1^	2009–2017	None	−0.7 (−0.9, −0.5) ^1^	−0.7 (−0.9, −0.5) ^1^
Hepatobiliary cancer	2009–2017			−2.8 (−5.5, 0.0) ^1^	2009–2017	None	−1.3 (−1.7, −0.8) ^1^	−1.3 (−1.7, −0.8) ^1^
	2009–2014	2014	+0.5 (−3.1, +4.3)					
	2014–2017		−8.0 (−15.7, +0.3)					
Colon cancer	2009–2017			−1.6 (−4.9, +1.8)	2009–2017			−1.4 (−2.9, 0.0) ^1^
	2009–2011	2011	+9.3 (−8.5, +30.5)		2009–2011	2011	2.2 (−5.4, +10.3)	
	2011–2017		−5.0 (−7.4, −2.6) ^1^		2011–2017		−2.6 (−3.8, −1.5) ^1^	
Stomach cancer	2009–2017	None	−2.0 (−3.2, −0.9) ^1^	−2.0 (−3.2, −0.9) ^1^	2009–2017	None	−1.3 (−1.8, −0.7) ^1^	−1.3 (−1.8, −0.7) ^1^
Pancreas cancer	2009–2017			−2.1 (−5.8, +1.8)	2009–2017	None	−0.7 (−1.6, +0.1)	−0.7 (−1.6, +0.1)
	2009–2014	2014	+3.7 (−2.3, +10.0)					
	2014–2017		−11.0 (−20.0, −0.8) ^1^					
Leukemia	2009–2017	None	−2.2 (−4.7, +0.3)	−2.2 (−4.7, +0.3)	2009–2017	None	−1.5 (−2.4, −0.6) ^1^	−1.5 (−2.4, −0.6) ^1^
Non-Hodgkin lymphoma	2009–2017	None	−1.4 (−4.0, +1.4)	−1.4 (−4.0, +1.4)	2009–2017	None	−1.1 (−2.3, +0.1)	−1.1 (−2.3, +0.1)

^1^ Significantly different from zero (*p* < 0.05). Abbreviations: APC indicates annual percent change; AAPC, average annual percent change; CI, confidence interval.

## Data Availability

No new data were created or analyzed in this study. Data sharing is not applicable to this article.

## Supplementary Materials

The following are available online at https://www.mdpi.com/2072-6694/13/4/657/s1, Figure S1: Distribution of specific cancer types among the included septic shock cases from 2009 to 2017 (3-year intervals).
