# Supplementary figures and images for: Short and Long-Term Mortality Trends for Cancer Patients with Septic Shock Stratified by Cancer Type from 2009 to 2017: A Population-Based Cohort Study
Source: Cancers (Basel). 2021 Feb 6;13(4):657. doi: 10.3390/cancers13040657 (PMC7931033; doi:10.3390/cancers13040657)

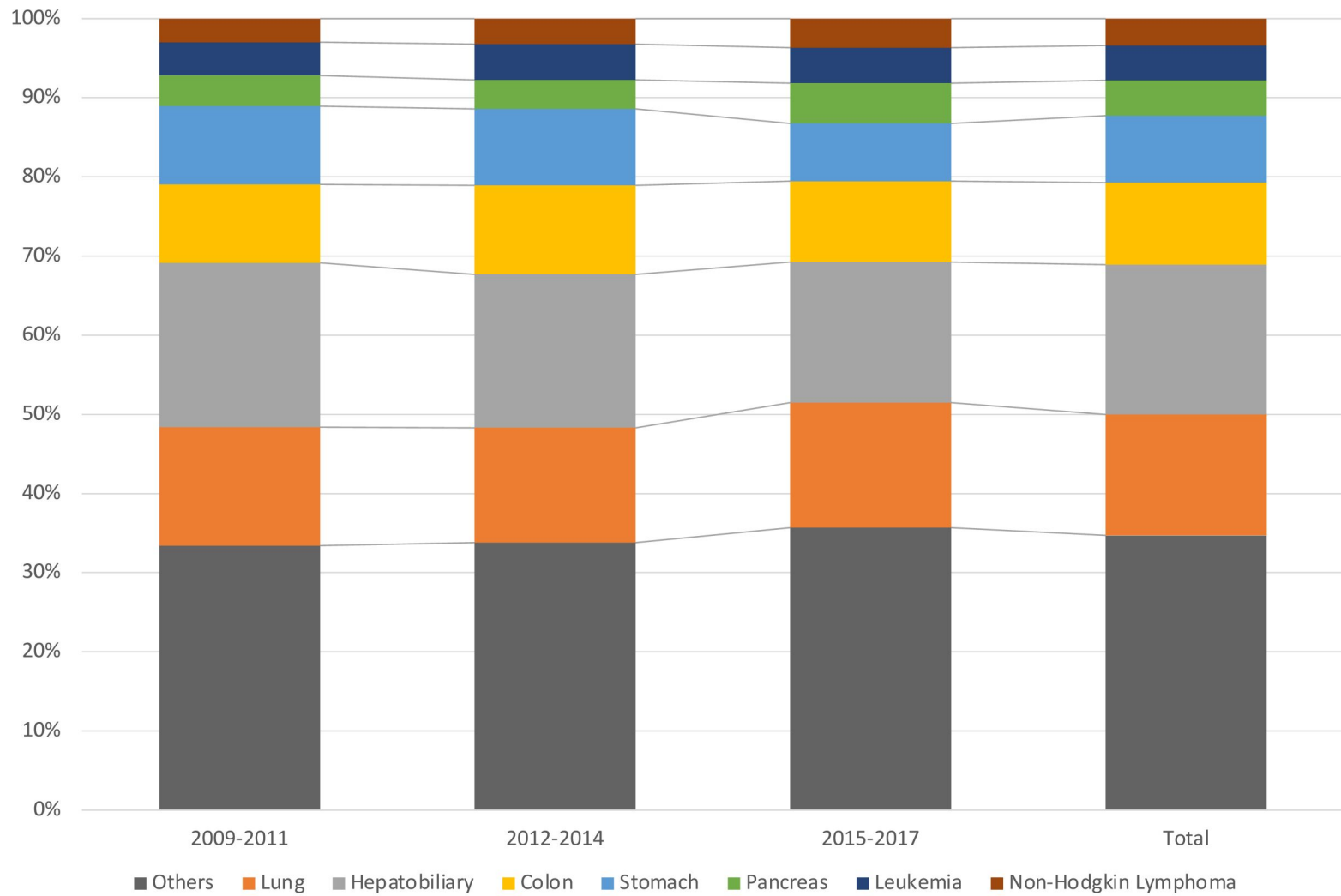

Supplement: Supplementary file 1 [file cancers-13-00657-s001.pdf]
